# A qualitative needs assessment of human trafficking in Ethiopia: recommendations for a comprehensive, coordinated response

**DOI:** 10.1186/s12939-020-1154-4

**Published:** 2020-03-17

**Authors:** Kristen R. Choi, Dana C. Beck, Munmun A. Khan, Sue Anne Bell, Lemlem Beza, Michelle L. Munro-Kramer

**Affiliations:** 1 University of California, Los Angeles School of Nursing, Los Angeles, CA 90095 USA; 2 University of Michigan School of Nursing, Ann Arbor, MI 48109 USA; 3 Health Policy Analyst, Atlanta, GA 30329 USA; 4 Addis Ababa University School of Medicine, Addis Ababa, Ethiopia

**Keywords:** Human trafficking, Ethiopia, Service needs, Needs assessment

## Abstract

**Background:**

Human trafficking is a global human rights violation that has profound health, economic, and social impacts. There has been little investigation of service needs and response options for human trafficking survivors in Ethiopia. The purpose of this study was to understand the potential service needs and response options for human trafficking in Ethiopia from multiple stakeholder perspectives.

**Methods:**

We conducted a qualitative needs assessment and used content analysis to analyze individual interviews with key stakeholder groups including service providers, academics, lawyers, and non-government organization (NGO) workers between the summer of 2015 – spring of 2016.

**Results:**

In total, 17 individuals participated and content analysis elicited four overarching themes related to post-trafficking needs, including mental health considerations, barriers and facilitators to providing survivor services, survivor service needs, and comprehensive care models.

**Conclusions:**

This qualitative needs assessment suggests that trafficking survivors may require professional and community services throughout their trafficking experiences, including medical care, economic and job opportunities, legal advocacy, and mental health services. Interventions should harness preexisting community strengths such as Ethiopia’s “*social healing system,”* health extension workers, and mobile technology. Future studies should explore tailored interventions and comprehensive models of care implemented within the pre-existing healthcare, social service, and community structures.

## Background

Human trafficking, or modern slavery, is a global human rights violation that occurs around the world and has profoundly negative impacts on human health, social welfare, and economic development [1, 2]. According to the United Nations Office on Drugs and Crime, human trafficking is comprised of three elements: (1) the act (recruiting, transporting, transferring, harboring, or receipt of persons), (2) the means (threat or use of force, fraud, or coercion), and (3) the purpose (exploitation including labor, sex, organ trafficking, slavery, child marriage, and other types of exploitation) [3]. The two major forms of human trafficking occurring around the world are labor trafficking and sex trafficking. Statistical measurement of prevalence has been difficult to capture due to the challenges of identifying those who have been trafficked, including the transience of the population, the variety of definitions, and the subjectivity involved in the interpretation of said definitions [4, 5]. Despite these measurement difficulties, organizations such as the International Labor Organization report that in 2016 approximately 40.3 million people are in modern slavery, including 20.9 million individuals who are victims of forced labor and 15.4 million individuals who are victims of forced marriage [6].

The United Nations Sustainable Development Goals adopted in 2015 specifically list as a goal the eradication of human trafficking through comprehensive, multilateral collaboration [7, 8]. In response, there have been attempts to address human trafficking by multiple organizations worldwide by means of creating and implementing public policies, capacity building, developing support for counter-trafficking measures, and improving support for those who have been trafficked [9]. While these large-scale, international collaborations are a critical step forward in addressing human trafficking, there remains a need for country-specific collaborations and solutions as well, given the wide array of historical, political, social, and cultural factors that affect trafficking and vary substantially by region. This paper specifically focuses on human trafficking in Ethiopia, where our team has worked extensively on partnered research.

### Human trafficking in Ethiopia

Ethiopia is a landlocked country in Eastern Africa within close proximity to the Middle East. It is the second most populous country in Africa [10]. Ethiopia is currently categorized as a low-and-middle income country; however, with significant growth it is poised to become a middle-income country within the next decade [10]. Ethiopia has continued to see growth in agriculture, industry, and service which has spurred a decline in poverty, particularly in urban areas [11]. Rural areas of Ethiopia are impacted by weather conditions unsuitable to agricultural activities, lower rates of secondary education among youth (14% versus 22% of rural youth ages 15–24 years old have a secondary education compared to those in urban areas), and lower rates of industry [11]. In Ethiopia, both migrants and residents are at risk for trafficking-related human rights violations driven by an exploitation of vulnerabilities for the men, women, boys, and girls who are trafficked [12, 13]. The popular media often focuses on Ethiopia as a transit location for women and girls being trafficked out of the country, but in reality, the problem is much broader in scope. Men, boys, women, and girls can all be exploited through various forms of both domestic and international trafficking [13, 14]. Human trafficking has been documented in begging, weaving, farm labor, and in brothels throughout Ethiopia [13]. Echoing these concerns, the 2018 United States Trafficking in Persons Report indicated that the Ethiopian government had not adequately addressed the problem of internal, within country trafficking, specifically lacking standardized protocols for frontline workers caring for survivors [15].

Research investigating human trafficking within and from Ethiopia indicates that trafficking often has severe consequences for survivors in all dimensions of their lives. Although studies in trafficked populations in Ethiopia are limited, case reports and small descriptive studies suggest that survivors frequently experience physical injuries, infectious disease, mental health disorders, shame, social stigma, and physical, sexual, and emotional abuse [16–23]. Often, finding post-trafficking support services and reintegrating back into communities are key challenges for survivors, with few resources available to aid survivors in this process [12]. As in many other regions of the world, services for human trafficking survivors are often pieced together from different agencies. There are no countrywide formalized, or coordinated processes of reintegration, networks of response, or systems of comprehensive service delivery to aid survivors of human trafficking in Ethiopia, all of which are needed to address the complex needs of trafficking survivors that are being observed [12]. In 2017, a non-governmental organization (NGO) working in Addis Ababa documented providing care to survivors of transnational human trafficking and there are other known care centers focused on in-country sex trafficking, but the care system remains severely underdeveloped with few known services for survivors outside of urban centers [15].

Furthermore, there are limited levels of awareness about human trafficking throughout the country, particularly in rural areas and among youth [24, 25]. One study focused exclusively on sex trafficking in northwestern Ethiopia found that 60% of participants had heard or read about sex trafficking and the most frequently reported source was television [24]. Ethiopia uses television, billboards, and word of mouth to increase awareness about human trafficking, but as these studies suggest, not everyone receives those messages. Given the lack of research in this area and the limited capacity of current survivor services, the purpose of this study was to conduct a qualitative assessment of the potential service needs and response options for human trafficking in Ethiopia. We sought to identify resources and delivery methods that key stakeholder groups—including service providers, academics, lawyers, and NGO workers—in Ethiopia believed would be most effective in meeting the complex needs of this vulnerable population.

## Methods

### Design

This study was a qualitative needs assessment that used content analysis with an aim of understanding the potential service needs and response options for human trafficking in Ethiopia from service provider, academic, lawyer, and NGO perspectives [26]. We chose a qualitative approach because human trafficking in Ethiopia is a relatively new, emerging area of research. A literature review revealed no intervention studies to date and few studies that have identified service needs and response options for this population [12]. This qualitative needs assessment study was the first step directed towards comprehensively identifying the needs of human trafficking survivors and eventual intervention development.

The needs assessment was conducted during two timeframes and in two locations: (1) interviews with service providers, academics, and NGO workers with expertise in human trafficking in the summer of 2015 in urban areas; and (2) interviews with an additional lawyer and service provider in the northern rural region of Ethiopia in spring of 2016. The data collected in 2015 were considered exempt from Institutional Review Board (IRB) regulation because it involved interviews in which human subjects could not be identified directly or through identifiers linked to the subjects. For the data collected in the spring of 2016 we received ethical approval from the University of Michigan Health Sciences and Behavioral Sciences IRB [HUM00113823] and Aksum University.

### Setting

The interviews were conducted in two regions of Ethiopia, around the capital city of Addis Ababa and in the northern region near Aksum. We selected these two interview sites to capture participants from both urban and rural settings where trafficking may occur in different ways.

### Sampling and data collection procedures

Purposive and snowball sampling were used to identify participants. We targeted participants from four key stakeholder groups: (1) service professionals (e.g., nurses, physicians, social workers), (2) academics, (3) lawyers, and (4) NGO personnel with expertise related to human trafficking. We chose these groups to gather information from experts with direct knowledge or experience with human trafficking and the cultural, political, and social context of Ethiopia.

Participants were contacted via email or telephone depending on which method of contact was available and invited to participate in the study. Potential participants were identified from online searches of published reports on human trafficking in Ethiopia, referrals from our in-country collaborators, and through snowball sampling. Inclusion criteria included: (1) Adults (age ≥ 18 years old); (2) English speaking (because post-secondary education in Ethiopia is conducted exclusively in English, the population of professionals we sought to sample was English-speaking); (3) self-identify as a service provider, academic, lawyer, or NGO advocate working with human trafficking survivors; and (4) available for a telephone or in-person interview in the summer of 2015 or spring of 2016. When participants responded positively to invitations, an in-person or telephone interview time was scheduled.

Data were collected using semi-structured interviews with participants in a location of their choosing (private or public interview locations were offered). Comprehensive verbal informed consent was obtained prior to data collection and all participants had the choice to opt out of audio-recording. These interviews were audio-recorded and transcribed and lasted approximately thirty minutes (range 20–40 min). Two interviewers conducted each session in English and asked follow-up questions as needed. One interviewer used a guide to ask the participant questions, and the other interviewer made field notes during the conversation and monitored the well-being of the participant due to the sensitive nature of the topic. Interview questions focused on the topics of: resources and services available and used by human trafficking survivors; what services should be available to human trafficking survivors; and how those in the health system could be involved in the prevention, education, and delivery of care for human trafficking survivors.

### Analysis

The interview transcripts and notes were coded by four members of the research staff (KC, DB, MK, MMK) using the constant comparative method, which is an inductive coding process for comparing and categorizing qualitative data [27, 28]. All transcripts were transferred to Microsoft Word and the research staff used color coding and comments to create categories and notes. First, each interview was read and analyzed independently by two coders using an inductive approach, which first involved an open coding process or allowing the codes to appear naturally without a pre-defined coding schema [27]. Next, the coders met to compare the resulting coding categories for agreement [27]. These open codes were compiled into a single analytical memo and were combined into four major categories [27]. Then two coders returned to the interview data to ensure that the major categories accurately represented all of the data. Once data saturation was reached, the themes were reviewed by all authors for validity [29]. Themes were derived directly from the data, and equal weight was given to the responses of all subjects in developing the themes.

## Results

In total, 31 individuals were invited to participate in the study. Twelve did not respond to participation invitations or were not available during the interview period and two declined to participate. The final sample was comprised of 17 participants. To protect their identities, limited demographics about the participants are presented in Table 1. All participants were native Ethiopians who read and spoke English and had a college education. Analysis of the qualitative data obtained from interviews with service professionals, academics, lawyers, and NGO personnel identified four overarching themes. These four overarching themes were: 1) mental health considerations, 2) barriers and facilitators to providing survivor services, 3) survivor service needs, and 4) comprehensive care models. The themes are expanded upon with additional details below.

**Table 1:** Participant demographics

Role/Profession	Number of participants	Gender	Age Range and Mean
Academic	8	Female = 3	28–49 (M: 34.63) years old
		Male = 4	
		Missing =1	
Service Professional	4	Female = 1	27–42 (M: 33.75) years old
		Male = 3	
Academic & Service Professional	2	Female = 0	Missing
		Male = 2	
NGO	3	Female = 2	35–48 (M: 40.67) years old
		Male = 1	
**Totals**	**17**	**Female = 6**	**27–49 (M: 35.06) years old**
		**Male = 10**	
		**Missing = 1**	

### Mental health considerations

The availability of mental health services was perceived to be inadequate for those survivors of human trafficking impacted by mental illness. Survivors of human trafficking were perceived to implicitly have mental illness following their trafficking experience. Multiple participants perceived that mental illness is generally under-resourced, under-treated, and under-reported in Ethiopia and situated the mental health needs of trafficking survivors within the overall context of Ethiopian mental health system challenges. As noted by one academic, *“In general, mental health services in Ethiopia are very limited. It’s not only for the human trafficking survivors but in general for every citizen [who] will have mental health challenges.”* There were concerns raised about the tendency towards nondisclosure of mental illness in Ethiopia regardless of an individual’s experience with human trafficking. As stated by one physician, *“I believe there is a great deal of underreporting and under-diagnosing of mental health issues in general.”* Participants also underscored the challenge of strong cultural stigma related to mental illness and worried that it could affect survivor perceptions of mental health services, as noted by an academic participant: *“...It’s kind of a taboo here. If you have a problem of mental [illness], the stigma that the society levels on you [makes it] very difficult for the people to seek mental health services...”*

In addition to the cultural stigma surrounding mental health, participants also discussed the cultural tendency to attribute mental illness to magic, witchcraft, or spiritual forces. The attribution of mental illness to non-medical causes was illustrated as determining a path to care that often began with religious or spiritual leaders rather than healthcare professionals and may delay survivor help-seeking for mental health care. As stated by a service provider: “*When you look at the flow of the patient, initially most of the patients before they came to hospitals or health facility [ies], they visit the religious and traditional healers in religious areas like [a] mosque or church first.”* Presenting for care at a health facility was seen as the last resort that people sought for mental illness, with the perception that trafficking survivors with mental health challenges would only be brought to healthcare providers if their needs escalated and became unmanageable by other means. According to participants, psychological symptoms are often expressed in terms of physical complaints, another factor that may delay or prevent survivors from seeking mental health care. This was echoed by one academic who stated that*, “much of mental illness [is] more expressed in terms of somatization.”*

### Barriers and facilitators to providing survivor services

The second theme to emerge among participants was about perceived barriers and facilitators to providing services to survivors of human trafficking. Participants seemed to describe two levels of barriers: individual and systemic. In the area of individual barriers, participants highlighted concerns related to the potential for survivors to be unaware of their needs or the possibility that they may qualify for assistance. Per one academic, “*one, there is a lack of awareness, secondly those victims do not come forward to expose their problem and seek advice.”* This is consistent with literature about human trafficking and other forms of gender-based violence around the world, many participants do not identify themselves as victims [30]. Participants suggested that survivors may face shame and isolation upon their exit from a trafficking situation when they could not provide their families and communities with the financial gain that is expected from those who work abroad. As one nurse stated:

“*It’s not only mental, it’s not only economical, what about the social? They need to reintegrate because they are not [the] only [one] who is going with expectations … their relative [s] and parents have expectations. If they don’t send the money in time, if they come [back] handicap [ped] with some illness, that means they will be a burden to their family.”*

These individual-level issues were seen as key barriers to survivor help-seeking.

In regards to systemic barriers, it was noted that broader societal issues such as poverty and high rates of migration are factors that may complicate the provision of services for survivors. Participants discussed the socio-cultural environment that they perceived poverty to create in Ethiopia, where in some areas people may consider migration their only choice for a chance at education or gainful employment, making them especially vulnerable to trafficking. An academic stated:

“*In some societies, migration of girls, intentional migration of girls to the Middle East was not part of the culture. There was no history of international migration to the Middle East. But before the ban, before three or four years, it has become a culture and everybody’s dream is to migrate and to come with some money. Even if you’re going to join the university or preparatory school, that is the only option, migration. Sometimes it is related to being unsuccessful with family schooling. Sometimes it is family poverty.”*

This participant’s reference to a ‘ban’ refers to Ethiopia’s attempts to halt migration to countries in the Middle East enacted in 2013 [31]. The overall challenge of managing trafficking amidst a culture of migration was echoed by another participant, who considered migration as a contributing factor to human trafficking to be an inevitable part of Ethiopia’s society: *“The government is of the view that we can stop migration. I say no. I think that we [can] better manage migration, but we can’t stop it.”* This participant viewed migration as a systemic, societal factor that was impossible to stop, but instead should be managed to facilitate prevention of human trafficking and provision of survivor services. Likewise, poverty and economic challenges were identified as key systemic barriers to trafficking survivors. Participants emphasized that trafficking survivors often are lured into exploitative situations out of economic desperation and the belief that they can make money abroad; as one participant stated, “*The first need is to be economically strengthened; if they were strengthened economic [ally] they may not be violated.*” Another participant described her surprise that survivors are focused on financial and economic challenges even in the midst of other serious needs upon return to Ethiopia:

*“At the beginning I was saying, ‘ … Their bodies are more important than money. All other abuses are more difficult than salary.’ But when they go, they have determined that they will be in all this pain to get money, come back and get money to do their own business. When they return, still they focus on economic issues.”*

The systemic presence of a culture of migration and poverty in Ethiopia were perceived to both contribute to risk for trafficking and to complicate service provision for survivors.

Participants identified a variety of cultural and country-specific strengths that may be facilitators to providing survivor services (Table 2). Facilitators to providing survivor services at the individual level included developing mobile or texting-based services to take advantage of Ethiopia’s expanded capacity for mobile phone access; creating opportunities for story-telling, narrative therapies, and peer connection within local communities; integrating spiritual, religious, and traditional healing practices into professional care; and developing and involving health extension workers in service provision in areas with limited availability of service professionals. Health extension workers are individuals that have limited training to meet healthcare needs in rural or underserved areas by providing education and basic services related to such conditions as hygiene and sanitation, family planning, and disease prevention and control. They are similar to community health workers and are more widely available in some underserved areas of Ethiopia than service professionals.

**Table 2:** Facilitators of survivor service provision

**Individual-Level Facilitators**	**Supporting Quotes**
Integrate religious/spiritual beliefs around mental illness and traditional healing practices (e.g., holy water) into professional care delivery	• *“They go to holy water place, and these traditional healers. And they say, at least some of them actually benefit from these interventions. These days there are some initiative to integrate the two approaches. Working together with the holy water people and the traditional healers.”*
Send text messages for patient tracking and retention in care; the trafficking population is highly mobile and difficult to retain in treatment, similar to the HIV population	• *“Some kind of retention mechanism; retention in care, in mental health care. One thing I suspect will be common from my experience working in the HIV care setting is that starting people on a given treatment, whatever that treatment may be, is not very difficult. Keeping them in that care is difficult. Given the fact that victims of human trafficking may be highly mobile, we have to make sure we create a way whereby they get care in the place wherever they go. Some kind of mechanism to find them would be SMS, or some kind of mechanism to track them.”*
Create opportunities for peer connections first, before offering professional services; shame and feeling alone can be barriers to help seeking and peers who have had shared experiences can help survivors be more receptive to services.	• *“Peer support, just to give them the opportunity to openly speak about their problems and so on, that’s a first support. Then after that they need another support, sustainable support.”* • *“Peer support is very important and one of the problems that potential migrants raise and even returnees raise. These abuses create a feeling of shame and they don’t clearly articulate what has happened to them. As a result, you know, potential migrants will not learn from them. “* • *“The peer model, I’m sure it would work because these are young people and most of the people I see have nowhere to turn to and to talk to. They have nobody to talk to.”*
Establish safe houses.	• *“There has to be safe houses and there has to be rehabilitation center where they can get immediate health service and a follow up for business engagement or enterprise. Whatever they want to do. But the problem is that we don’t have such centers.”*
Offer narrative therapy.	• *“When you deal with this narrative exposure therapy, you give them the opportunity to write their narratives and they would have a written statement at the end of the therapy. It’s something that is dramatic and it gives you closure.”*
**System-Level Facilitators**	**Supporting Quotes**
Ensure that interventions are a community effort; involve educators, academics, and policymakers in rehabilitation/reintegration efforts	• *“The issue will not be left for only mental health professionals. The community should have to participate. The policymakers should have to be there. We who are teaching at university level should have to be there. The healthcare providers should have to be there. There should be an inter-collaboration.”* • *“If we want to implement something else here unless it is aligned with the government policy and there is a favorable policy here, you can’t do anything else even though we came up with the huge amount of money.”* • *“There should be a concerted effort. It should not be piecemeal. It should be concerted and sustainable.”*
Strategize about most likely “entry points” into service systems; identify where are survivors most likely to interface with professionals and offer services there (e.g., airports, healthcare, community health workers)	• *“When we were talking about entry points, are legal services really an ideal entry point? Going to the legal services may not be the first thing that comes to mind or, they don’t want to go public with that. It’s a kind of shame, so they just keep it there. The health services I would say could be you know, a first entry point.”* • *“The area that you want to work from is the airport. The people that in that institution may expose human trafficking.”* • *“If these health extension workers are getting adequate training about trafficking including sex trafficking, we can deliver enough information or we can distribute enough information about the awareness of trafficking for the wider community.”*
Locate survivor services health, legal, economic, and social services at one center; use “one-stop shopping”	• *“The one-stop shopping idea is something you have to very seriously consider. If there is a scheduled service delivery to victims of human trafficking, you can have the legal advisors come to the clinic or the healthcare providers go to the place where legal advice is provided. But you have to make that available at one stop so that one stop shopping can be entertained.”*
Activate local community support structures; Ethiopia has a rich “social healing system,” described as including the following entities: • **Kebele**: A small, localized administrative unit; a neighborhood or ward; • **Mahiber** (mahibere): Amharic for ‘association;’ a cultural development association; • **Iquib** (iqub,equb): A sustainable rotating funds association established by a small group of people in a community; and • **Idir**: A burial society association established in small communities to raise money during emergencies, particularly after a death in a community.	• *“There are these associations in every village. Everyone would be involved in idirs. It is voluntary, but usually everybody is involved. And the responsibility of these idirs is to take care of you in your time of need. These things, idirs, have been with us for, I don’t know, maybe for hundreds of years. These are very deep-rooted, very good resources to think about.”* • *“The most resource that we have is the social ties that we have. It’s not like the western type, that is more individual. Nowadays due to globalization and due to fast urbanization, that type of thinking is coming but as a culture we have society norms that support each other, even though we are poor and even though we don’t have the money. We need to capitalize that. We need to use that as a good resource.”* • *“The local government together with community-based association; there are lot of community based associations … .so people support each other during hard times. During weddings, funerals, and during economic bankruptcy or some problems, they support each other. There are self-help organizations at the community level. If these returnees are honest enough or they clearly express their needs to the local community, even though their parents are poor, destitute, some better off members of their community can support”* • *“The social healing system … that is part of social medicine.”* • *“One of the strengths we have, one of the cultural strengths we have is the social relationships, social interactions. Social support.”*

At a system level, participants suggested several additional facilitators to providing survivor services. These included locating services or screening in non-traditional settings (e.g., airports) where survivors are likely to be encountered; co-locating multiple kinds of professional services; ensuring that interventions are community-based but also involve educators, academics, and policymakers; and activating Ethiopia’s local community support structures (described as a “*social healing system*” by one participant). These include cultural development associations (Mahiber, Mahibere), rotating fund associations (Iquib, iqub, equb), burial society associations (Idir), and local neighborhoods or wards (Kebele). Such preexisting community support structures may be well-suited to providing direct survivor support or facilitating engagement in professional or community-based services,

### Survivor service needs

The third theme to emerge was survivor service needs, which included education about trafficking risks and trafficking prevention interventions, mental and physical health care, job skills training, legal support, and reintegration. Participants identified that there was a need for education directed towards both recovery (e.g., availability of survivor services) and prevention (e.g., risks of illegal migration and push and pull factors related to human trafficking). One physician participant noted that this education and prevention could be integrated in pre-existing models: *“Let’s not miss the opportunity. When we see a mother, does she have kids, are they vaccinated? Let’s embed [prevention education] with family planning … a level awareness [and] to have that consciousness.”* Participants focused on outreach by noting that NGOs and the government should provide educational awareness about human trafficking and that returnees could help with this awareness and education.

In addition, all participants discussed addressing mental and/or physical health care needs as a priority. Service professionals and NGO personnel focused on survivor needs such as counseling. One physician stated,

*“...Starting from health, I would recommend counseling, counseling is the best thing that they need right now. After that, of course there are also other health issues, the sexual abuse, and if they have contracted any kind of transmitted diseases, of course they need immediate health service [s].”*

Another participant added: *“Some of them may be physically injured and others are those who lost their legs, hands, who are amputated, who are face is disfigured. And again, most of them are sexually abused, so there can be possibility of getting Human Immunodeficiency Virus (HIV).”*

Participants also emphasized the importance of incorporating job skills training into survivor services. As one academic noted, “*… they faced various forms of sexual abuse, physical abuse, and degrading and insulting and all different forms of abuse. But they extremely feel the financial pain, they extremely feel the salary denial …*” Often, the reason individuals choose to migrate and subsequently find themselves being exploited in trafficking situations is because of poverty and lack of economic opportunity in Ethiopia. As such, financial and employment needs appear to be at the forefront of their minds upon returning, sometimes more so than immediate physical and mental health concerns that might be mistakenly perceived by support personnel to be the survivor’s first priority.

It was also noted that the stigma for survivors of human trafficking and the lack of awareness as to the kinds of legal services that may benefit them are prohibitive to survivors receiving legal support. All participants specifically referenced the need for legal services for gender-based violence and safe migration but did not specifically allude to what these services might look like. They did discuss a need for peer support from others who had experienced trafficking, provisions for immediate physical safety (e.g., shelter, food, clothing), and longer-term stabilization (e.g., housing, employment, financial support, skills training, stigma reduction) as modalities for reintegration. An academic summarized this sentiment by stating,

“*First they have to be socially integrated. Then the physical, psychosocial, even the mental than the physical health. So step by step, we need to address all dimensions of health problems related to the returnees. But the first priority should be given [to the] social well-being of these returnees.”*

This perspective illustrates the importance of “*social healing systems*” for trafficking survivors in Ethiopia given their cultural context.

### Comprehensive care models

There was a common theme among all participants that offering care for survivors of human trafficking through a comprehensive model would be the optimal approach. Participants emphasized the importance of working together in terms of system change and integrated interventions rather than piecemeal solutions. As one physician stated,

*“I think the most important thing is to work in terms of systems, rather than crisis interventions...So we have to take into consideration, every step of intervention. So when they come here, what about providing them food, and shelter, and other things that are really necessary. Then what about screening? What type of instruments should we use? Then, to whom should we send these patients?”*

This sentiment was reiterated by other participants. They recommended community-based interventions and utilizing community resources and social structures to deliver interventions, which include awareness and prevention efforts. A service provider suggested, *“to tackle or to defend for [this] challenge, so at [the] community level some awareness in intervention is needed in [the] community to prevent that.”*

## Discussion

This study found four overarching themes reported from service professionals, academics, lawyers, and NGO personnel with expertise in human trafficking. These four overarching themes included mental health considerations, barriers and facilitators to providing survivor services, survivor service needs, and comprehensive care models. There was a significant focus on mental health services. Participants spoke about the mental health needs of trafficking survivors and also about the challenges of providing mental health services in a general context of scarcity of mental health resources. Mental health is often stigmatized in Ethiopia and other regions of Africa [32, 33], which can be a barrier to help-seeking. Given Ethiopia’s mental health context, there may be limited resources for those with mental health problems including trafficking survivors. The number of mental health facilities and clinicians in Ethiopia is limited; the Ethiopian Ministry of Health estimates that there are 40 psychiatrists and 461 psychiatric nurses in the country serving patients at only a few inpatient and outpatient facilities designated for mental health [33, 34]. While there is very limited empirical evidence on the mental health needs of human trafficking survivors in Ethiopia, past research with migrant returnees has documented mental health disorders among 27.6% of the sample [35]. This research supports the perspectives of our participants, who also noted that in Ethiopia, mental health problems are often experienced as somatic symptoms [35].

In order to address the mental health needs of trafficking survivors, it is important to increase the number of service providers trained to provide mental health services. The Ethiopian Mental Health Strategy began implementing the World Health Organization Mental Health Gap Action Plan (mhGAP) guidelines in 2012 [36]. This included programs to bolster the integration of mental health services into primary care along with an increase in the clinicians and health workers trained in the provision of mental health services [36]. In rural Ethiopia, specially trained community-based rehabilitation workers and supervisors were effective in delivering a community-based rehabilitation program for people diagnosed with schizophrenia and their families, showing improvements across multiple individual, family, and community domains [37]. The increase in available services for mental health in Ethiopia is likely to benefit survivors of trafficking who may have mental health needs. Incorporating education about human trafficking into the training of mental health clinicians and health extension workers may improve identification of and intervention with trafficking survivors, along with increasing awareness at the community level through existing structures.

Despite the recognition among study participants that trafficking survivors have needs, there are a number of interpersonal and systemic barriers that prevent survivors from meeting those needs. A brief published by the United States Department of Health and Human Services noted that potential barriers include a lack of knowledge and understanding about trafficking among survivors and service providers; the availability, appropriateness, access, and length of services may not meet the needs of survivors; and a lack of coordination among service providers [38] Our study participants identified similar barriers. There has been a significant focus on healthcare providers and training them to identify and respond to survivors of human trafficking in the United States and around the world [39–42]. Although much of the academic literature has focused on barriers in the United States, it is likely that similar barriers exist around the world in regards to both interpersonal and systemic challenges. Future interventions for human trafficking survivors in Ethiopia may need to include components addressing these barriers to providing survivor services.

The results of this study also highlight the need to build on pre-existing services when considering the service needs of human trafficking survivors. For instance, participants noted that health extension workers in Ethiopia could be promoting information about the consequences of trafficking during their interaction with community members. This type of intervention would require minimal training and would engage a group that is already trusted by the community, as has been done with a mental health intervention in rural Ethiopia [43]; however, there is the potential that this might overburden health extension workers as was suggested in a review of community health workers providing services to survivors of sexual violence [44]. Integrative rather than additive solutions would be ideal. Participants also highlighted the need to bring together professionals focused on physical healthcare, mental healthcare, legal needs, and job skills training into one location. Currently many of these professionals work in silos within their own disciplines. There is a need to develop innovative solutions to bring together a diverse group of professionals that can meet the needs of human trafficking survivors in a holistic manner in a designated setting. This could start as a one-day clinic at a pre-existing site and expand based on survivor needs.

As noted in the literature and supported by this qualitative needs assessment, trafficking survivors are in need of a diverse array of legal, social, and healthcare services throughout their trafficking experience and after trafficking occurs [38, 45, 46]. An established conceptual model of human trafficking and health outlines stages in the trafficking life course from recruitment to exploitation to re-integration, and services should target each stage of this model [47]. Although our study participants focused primarily on post-trafficking services, concerns about issues that drive trafficking and experiences of disease and injury during trafficking experiences were frequently mentioned. For instance, prevention activities focused on increasing awareness about trafficking would be most useful before recruitment begins, as survivors may not necessarily be familiar with or identify with the concept of human trafficking and therefore may not identify as a victim when they experience it. Post-trafficking, job skills training and mental and physical healthcare may be most relevant. Despite the recognition that human trafficking survivors have diverse needs, there is a dearth of literature about comprehensive care approaches. As such, there is a need to develop and evaluate interdisciplinary approaches to delivering survivor service needs that address their complex care needs across the stages of human trafficking.

Despite the harmony in discussing the service needs of human trafficking survivors among all participants in our needs assessment, there appeared to be some important topics that were omitted during these conversations including gender considerations and the phenomenon of domestic trafficking. These issues are commonly cited in the literature on trafficking in Ethiopia and in other countries, but were not discussed extensively by participants in our sample [5, 12]. Throughout the interviews with advocates and service providers, we noted a focus on women and girls as survivors of international human trafficking with little attention to how trafficking affects boys and men, despite the International Labor Organization documenting that approximately 42% of forced labor victims are boys and men [6]. This omission demonstrates the complex gender dynamics of trafficking and suggests that there may be educational needs at the professional level surrounding assumptions or stereotypes about who is a victim of trafficking (i.e., assumed to be primarily women and girls). Feminist frameworks may be useful for this future work given the high prevalence of gender discrimination and gender-based violence associated with human trafficking, and education about trafficking should include attention to gender-specific needs of survivors.

Secondly, participants focused many of their responses and stories/anecdotes about trafficking on people going to the Middle East. While migration and trafficking from Ethiopia to the Middle East has been cited throughout the literature [48–50], the focus on international trafficking seemed to ignore the domestic trafficking occurring within Ethiopia. The United States Department of State has documented both sex and labor trafficking throughout Ethiopia [13]. This focus on international trafficking may have also impacted the participants’ responses about the types of services that survivors need. For instance, there have already been campaigns around Ethiopia to alert individuals about the risks of migration and human trafficking to the Middle East; however, there has been limited focus on domestic trafficking [48]. Future intervention and policy work should include a specific focus on domestic trafficking.

### Limitations

This study was conducted among a relatively small group of self-selected respondents who were willing to discuss human trafficking in Ethiopia, by a collaborative team based in the United States and Ethiopia. The representativeness of the sample is limited due to convenience sampling. Respondents were referred from local contacts, announcements, and then through word of mouth (snowball sampling). All interviews were conducted in English. Although our target population of professionals was expected to be fluent in English because of educational requirements in Ethiopia, those for whom English was a second language or those who felt uncomfortable speaking English may have refused participation in the study. Our study may have left out some stakeholder groups (e.g., government officials, transportation workers, employers) that may have valuable perspectives on post-trafficking service delivery in Ethiopia, and as such, future research should explore an expanded range of stakeholder perspectives.

Despite these limitations, this study provides a broad, initial assessment of the potential service needs and response options for human trafficking in Ethiopia and provides directions for service delivery, research, and policy action with this population. We sampled participants from four important stakeholder groups, and future studies may consider incorporating survivor voices in studying human trafficking. The study included participants from both rural and urban areas, and we found consistent evidence across all stakeholder groups and settings for the themes described above.

## Conclusion

This study illuminates the potential service needs and response options for human trafficking survivors from the viewpoints of diverse stakeholders in Ethiopia. Our results suggest a need for medical care, economic and job opportunities, legal advocacy, and mental health services, with a particular priority focus on mental health. Interventions should be developed at both the individual level and the system level, should be integrative and comprehensive, and should harness preexisting community strengths such as Ethiopia’s “*social healing system,”* health extension workers, and mobile technology. Future work should build on the findings of this study by assessing the needs of larger and more diverse samples (including human trafficking survivors), focusing on domestic as well as international trafficking, and developing and testing comprehensive interventions for the Ethiopian context.

## Data Availability

Data sharing is not applicable to this article as no datasets were generated or analyzed during the current study.

## Abbreviations

HIV: Human Immunodeficiency Virus
IRB: Institutional Review Board
mhGAP: World Health Organization Mental Health Gap Action Plan
NGO: Non-Governmental Organization
